# Primary extraskeletal osteosarcoma of sigmoid mesocolon: a case report and a review of the literature

**DOI:** 10.1186/s12957-021-02337-9

**Published:** 2021-09-03

**Authors:** Xinyang Nie, Weihua Fu, Chuan Li, Li Lu, Weidong Li

**Affiliations:** 1 The Graduate School, Tianjin Medical University, Tianjin, China; 2 Department of General Surgery, Tianjin Medical University General Hospital, 154, Anshan Road, Heping District, Tianjin, 300052 China

**Keywords:** Mesentery, Sigmoid mesocolon, Extraskeletal, Extraosseous, Osteosarcoma

## Abstract

**Background:**

Extraskeletal osteosarcoma (ESOS) is a rare mesenchymal malignancy, which produces osteoid, bone, or chondroid material and is located in the soft tissue without attachment to skeletal bones and periosteum. One of the things that ESOS originated from mesentery is much rarer.

**Case presentation:**

A 75-year female had a history of pain in the left lower abdomen for more than 4 months. Abdominal computerized tomography (CT) and magnetic resonance imaging revealed a large, irregular, and solid-cystic mass (largest diameter was 11.5 cm). The tumor was radically removed during an open operation. It was composed of abundant osteoid and polyhedral-shaped tumor cells with high atypia and high mitotic activity microscopically. The final pathological diagnosis was osteoblastic osteosarcoma, arising from the sigmoid mesocolon with negative margins. A 9-month follow-up by CT exhibited signs of peritoneal metastasis.

**Conclusions:**

Given the rarity of cases of mesenteric ESOS, diagnosis mainly depended on pathology findings or should be taken into consideration when the mesenteric mass was found. Its most effective treatment had not been determined, with surgical excision being generally accepted. Ensuring negative surgical margins may be an important factor affecting prognosis.

## Background

Extraskeletal osteosarcoma (ESOS) is a rare mesenchymal malignancy that usually occurs in the fifth or sixth decades of life, first described in 1941 by Wilson [1]. Generally, the tumor produces osteoid, bone, or chondroid material and is located in the soft tissue without attachment to the skeletal bones and periosteum [2], most frequently in the deep soft tissues of lower extremities, as well as in the upper extremities and retroperitoneum [3]. Low incidence of ESOS has been reported, accounting for only 4% of osteosarcoma and approximately 1% of soft tissue sarcoma [4–6]. ESOS arising from mesentery is extremely rare. Radical surgical resection remains the main treatment for ESOS [7]. Here, we describe a case of primary ESOS arising from sigmoid mesocolon.

## Case report

A 75-year-old woman with no history of malignancy was referred to our hospital in August 2020 after experiencing pain in the left lower abdomen for more than 4 months. No history of trauma, previous radiation, or a family history of genetic diseases was identified. There was no history of dark or bloody stools, but she reported a recent change in bowel habits lasting nearly 2 months which was caused by transient constipation that led to frequent use of laxatives. She had a 3 kg weight loss in the preceding months.

**Fig. 1:**
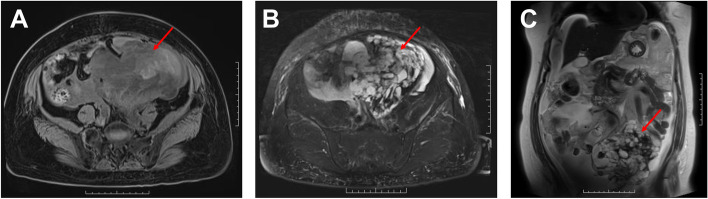
Magnetic resonance imaging: a large, irregular, multilocular, solid-cystic, and complex signal mass shadow, which is closely associated with the intestine (arrows). The liquid level can be seen locally inside. The solid part was isointense on both T1 and T2 images. (**A** T1 imaging, transverse plane; **B** T2 imaging, transverse plane; **C** T2 imaging, coronal plane)

Physical examination revealed a protuberant abdomen with a large, hard, nonpulsatile but painless mass in the left lower abdomen. Cardiovascular and respiratory examinations were unremarkable. Laboratory findings including serum electrolytes, hepatic functions, and renal functions were within normal limits, as well the serum alkaline phosphatase: 60 U/L (40–150 U/L). Standard blood examination showed a decreasing blood count (3.09 × 10^12^/L (3.80–5.10 × 10^12^/L)) and hemoglobin concentration (92 g/L (115–150 g/L)). Tumor markers such as AFP, CA199, HCG, HE4, and CEA were all normal, but CA125 was markedly elevated: 585.60 U/mL (0–35.0 U/mL). Following abdominal ultrasonography, a solid-cystic and space-occupying mass and blood flow signal can be seen inside. An abdominal computerized tomography (CT) scan revealed a mass adjacent to the left uterine adnexa area and closely related to the sigmoid colon, along with multiple lymph nodes in the pelvic and abdominal cavity. Magnetic resonance imaging revealed a large, irregular, multilocular, solid-cystic, and complex signal mass shadow. The solid part was isointense on both T1 and T2 images. Part of the mass was obviously hyperintense on DWI (Fig. 1). It was more likely to be considered as malignant mesenchymoma.

**Fig. 2:**
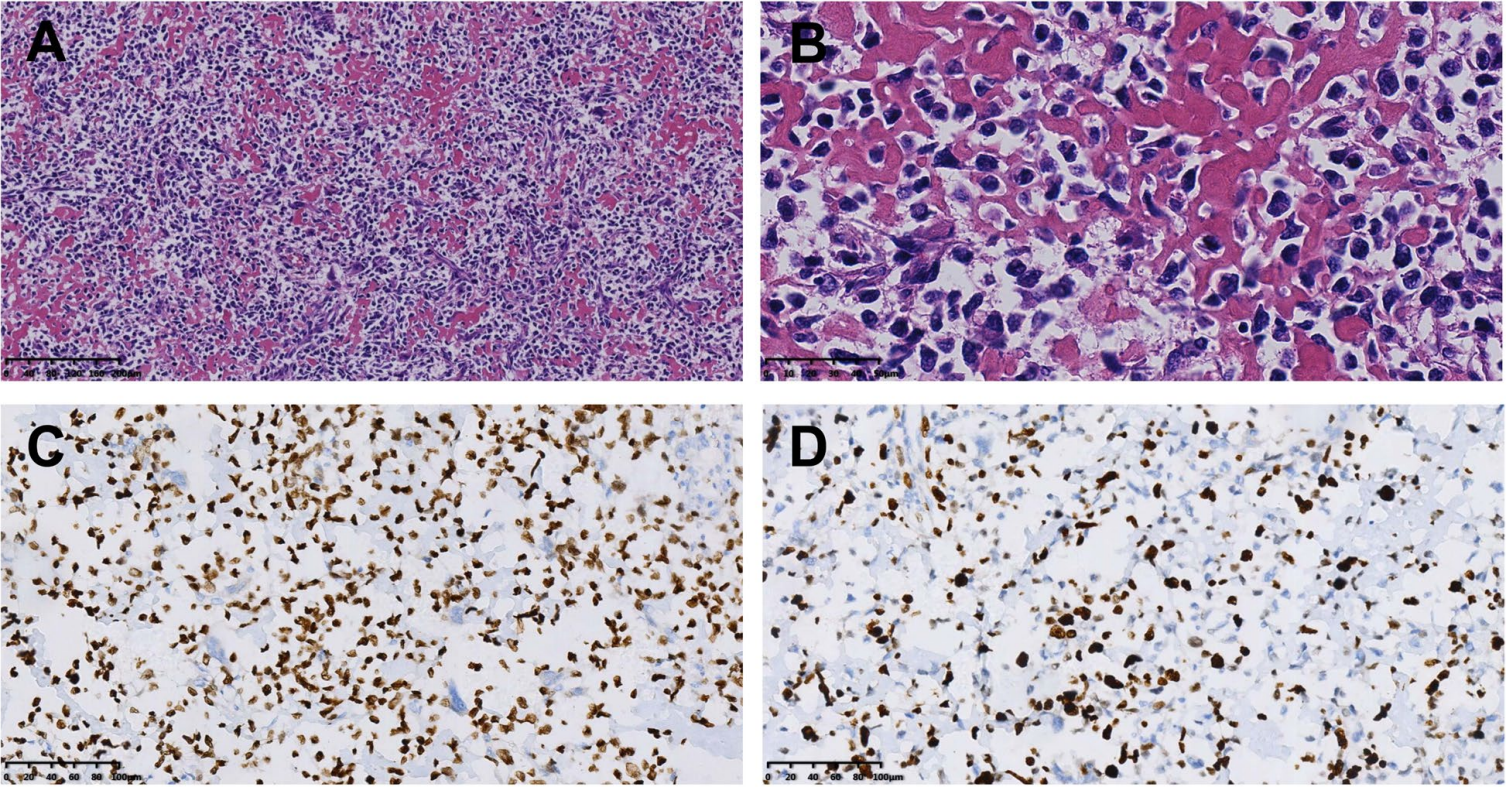
Histologic sections of the tumor: **A**, **B** HE-stained section: microscopically, there are many polyhedral tumor cells and abundant osteoid. The eosinophilic osteoid matrix could be found intimately admixed with the highly atypical tumor cells, presenting focal deposition (**A** original magnification × 100; **B** original magnification × 400). **C** Immunohistochemical staining reveals the tumor cells is positive for SATB2 (original magnification × 200). **D** Immunohistochemical staining reveals the Ki-67 positive rate is about 60% (original magnification × 200)

**Fig. 3:**
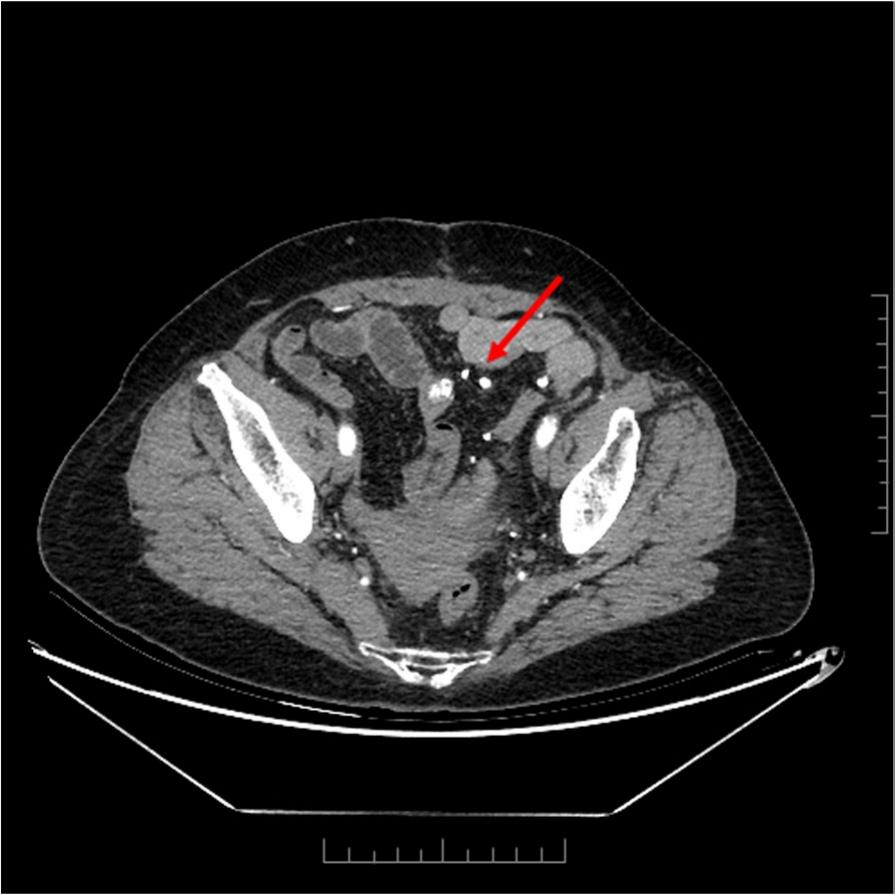
Abdominal CT-scan imaging: multiple new calcified masses found around the descending colon and the anastomotic, which showing irregular reinforcement, suspicious for metastasis (arrows)

During an exploratory laparotomy, a large solid-cystic mass was identified in the mesocolon of sigmoid with invasion into the sigmoid and small intestine. It became fixed on the posterior abdominal wall, accompanied by multiple ruptures and active hemorrhage on the surface. Multiple small hard nodules were found in the small bowel mesentery and sigmoid mesocolon. The tumor was resected en bloc with the sigmoid, ileocecal junction, part of the small bowel, bilateral fallopian tubes, and ovaries. The resected tumor was 11.5 cm × 7 cm × 6.5 cm in size. In addition, the other small lesions in the mesentery were completely resected. The tumor was heterogeneous on a microscopic level. The other small masses were demonstrated as focal ossification nodules. The tumor was composed of polyhedral-shaped tumor cells and abundant osteoid. The tumor cells exhibited high atypia, high mitotic activity, and atypical mitotic morphology. The eosinophilic osteoid matrix could be found intimately admixed with the tumor cells, presenting focal deposition. By immunohistochemistry, the neoplastic cells were positive for Vimentin, SATB2, Bcl2, SDHB, and CD99, but negative for cytokeratin, epithelial membrane antigen, desmin, CD117, CD34, Dog-1, and S-100. Part of them was positive for smooth muscle-actin and CD68, and Ki-67 positive rate was about 60% (Fig. 2). Combined with pathological findings, they did not support gastrointestinal stromal tumors, liposarcoma, or epithelial neoplasms. The final pathological diagnosis was osteoblastic osteosarcoma, arising from the sigmoid mesocolon with negative margins and no lymph nodes or blood vessel invasion. The patient was advised to receive chemotherapy after the operation, but she refused. After the diagnosis was established, a whole-body bone scan revealed no evidence of osseous metastatic disease. Therefore, the sigmoid mesocolon was considered the primary lesion. The patient was reviewed at 9 months postoperatively. CT showed multiple new calcified masses around the descending colon and the anastomotic, showing irregular reinforcement. They are considered metastatic lesions (Fig. 3).

## Discussion

Extraskeletal osteosarcoma (ESOS), also known as soft tissue osteosarcoma, is a rare malignant neoplasm that produces osteoid, bone, or chondroid material but lacks bone or periosteum involvement. Although the first report was described in 1941 [1], few cases have been reported so far [2]. ESOS is most frequently found in the lower extremity, particularly in the deep soft tissue of the thigh (42–77%), followed by the upper extremity (12%). It has also been reported that it was found in the retroperitoneum (12%). Other relatively rare sites have been previously reported, including the larynx, kidney, esophagus, small intestine, liver, heart, urinary bladder, parotid, and breast [2, 4]. In contrast to skeletal osteosarcoma, which always occurs in patients in the first three decades of life, most ESOS occurs in the fifth and seventh decades of life and at a mean reported age of 60 years [6, 8]. Males are slightly more than females, with a ratio of 1.9:1 [7, 9]. Controversially, there were also statistics indicating that the male predominance observed in primary osteosarcoma did not exist in ESOS patients [2]. While the exact cause of the ESOS is unknown, some reports revealed that it could be related to radiation, such as previous exposure to X-rays and radioactive thorium dioxide, or at least 4 years following high-dose radiation therapy [10]. Besides, some reports revealed that 12 to 30% of patients had experienced trauma, and some cases could occur after ossifying myositis [11, 12]. However, in our case, the patient had no prior history of trauma or radiation. The most common symptoms of ESOS included a painful or painless mass that grew slowly and progressively in the abdominal cavity. Generally, the mass was quite large when the patient sought treatment. If the mass invades the bowel, changes in defecation characteristics may occur, including constipation and blood-tinged stool. It is visible on ultrasonic, CT, and MRI as a large soft-tissue mass with no osseous involvement [3, 7, 11, 13, 14]. ESOS is not specific on imaging; in some cases, the radiological features described are a calcified mass on CT, but in our case, it is a solid-cystic mass [3, 7]. On MRI, the lesion is slightly hyperintense to muscle and also nonspecifically on T1-weighted imaging and exhibits high signal intensity on T2-weighted imaging, which contrasts with our case [15].

ESOS should be diagnosed using a combination of clinical manifestations and radiographical and pathological findings and only after excluding the possibility of a primary bone tumor or bone tumor metastatic to soft tissue [4]. Combined with clinical and imaging findings, it is necessary to differentiate it from liposarcoma, gastrointestinal stromal tumor, or hemangioma with calcification. For atypical clinical and radiographic manifestations such as the patient in our case, pathology may be the final diagnostic criterion, particularly for ESOS in the abdominal cavity. The histological differential diagnosis included de-differentiated liposarcoma with heterologous differentiation, malignant peripheral nerve sheath tumor, undifferentiated high-grade sarcoma, and carcinosarcoma. Consistent with WHO classification of tumors, ESOS was diagnosed by the pathologist based on the appearance of osteoid matrix and osteoblast-like tumor cells, the absence of adipocytic, myogenic, or neurogenic tumor differentiation, and the absence of de-differentiated or highly differentiated liposarcoma components on cross and microscopic examination of the specimen [16]. Pathological subtypes of ESOS can be divided into six types. One of the most common is the osteoblastic variant, such as in our case with abundant osteoid. Outside of that, chondroblasts, fibroblasts/pleiomorphic malignant fibrous histiocytoma-like cells, telangiectasis, small cell, and mixed types are present [4, 8].

Surgery is the main treatment for ESOS. Depending on differences in location, range, and development of the tumor, a simple resection, wide resection, or radical resection can be selected. Besides, preoperative radiotherapy and adjuvant chemotherapy are available to treat ESOS. According to statistics, expanding the scope of surgery can reduce the local recurrence rate but had no significant effect on prolonging the survival time [9]. According to the current situation, chemotherapy regimens and their effects on ESOS remain controversial. Ahmad et al. [17] reported that in 60 ESOS patients, 27 patients received with doxorubicin-based chemotherapy with an effective rate of 19%. Wang et al. [8] reported that most cases received methotrexate, adriamycin, and cisplatin-based chemotherapy regimens. A minority of patients received therapy with adriamycin or ifosfamide. However, there have been no survival benefits between different chemotherapy regimens or those who received chemotherapy and those who did not. Besides, when patients cannot accept surgical treatment, tolerate high dose chemotherapy, or have advanced disease, palliative radiotherapy may be considered. Preoperative or postoperative radiotherapy has been demonstrated to be beneficial in reducing the volume of tumors and local recurrence, without specific improvement in overall survival or progression-free survival and no difference in death due to disease or event-free survival [2, 8]. Radiotherapy is critical to improving overall survival in patients who cannot achieve negative surgical margins [8]. ESOS has a poor prognosis regardless of the tumor’s origin or location. ESOS has a high risk of local recurrence and distant metastasis. When the results of multiple reports were combined, the local recurrence rate was approximately 18–19% and distant metastasis was 37–38% [2, 17]. According to the reports, approximately 39% of patients died within 3 years of diagnosis [2], and approximately 75% died within 5 years of diagnosis [13]. Tumor size is a significant prognostic factor, as patients with tumors larger than 5 cm have worse clinical outcomes. Bane et al. [4] reported that the mortality rate associated with the disease for patients was about 14.3% (1 of 7 patients) for tumors smaller than 5 cm, but was 87.5% (14 of 16 patients) for tumors larger than 5 cm. Besides, positive margins following operation are an important factor that affects overall survival and local recurrence. Tumors with positive margins exhibit a higher risk of local recurrence and a lower 5-year survival rate. For patients with non-metastatic disease, the 5-year local control rate was about 89%, with no significant difference between positive and negative margins. The 10-year local control rate remained unchanged with negative margins, but reduced significantly with positive margins [8]. In the presented case, the patient was an older woman with a large tumor (> 5 cm in size) and was not receiving radiation and chemotherapy treatment. Although negative surgical margins were guaranteed, metastases were considered combined with CT findings 9 months after surgery. Even if the patient does not present with any symptoms postoperatively, predicting the prognosis remains a challenging task.

We summarize the reports of ESOS of mesentery that have been published in English to date (Table 1). It includes the patient’s basic characteristics, the tumor’s condition, treatment, and prognosis during initial diagnosis [3, 7, 13, 14, 18–22]. The average age of the ten patients (5 males) was 57 years (range, 39 to 75 years). Seven patients had tumors larger than 10 cm. They all underwent surgery, but only three accepted chemotherapy. By comparison, no significant improvement in prognosis was observed. In conclusion, this report illustrates ESOS arising from sigmoid mesocolon and should be considered in the differentials diagnosis of intraabdominal malignant mesenchymal tumors. The optimal treatment for mesentery ESOS remains a challenge.

**Table 1:** Literature review of ESOS of the mesentery cases

	Author (year)	Sex	Age	Size (cm)	Surgery	Adjuvant therapy	Margin	Prognosis
1	Fine G (1956) [18]	M	39	–	Y	–	–	Dead (55 days postoperatively)
2	Shirazi P H (1973) [19]	F	56	–	Y	N	–	Dead
3	Choudur HN (2005) [3]	M	45	15	Y	Doxorubicin cisplatin	–	Alive
4	Heukamp LC (2007) [13]	M	61	20	Y	Doxorubicin cisplatin cyclophosphamide ifosfamide	–	Dead (10 months postoperatively)
5	Lee KH (2007) [14]	M	67	18	Y	Ifosfamide adriamycin	–	Dead (4 months postoperatively)
6	Hussain MI (2011) [20]	M	40	13	Y	–	–	–
7	Oh SJ (2017) [21]	F	70	15	Y	N	–	Dead (2 months after discharge)
8	Van den Broek (2018) [7]	F	71	14	Y	N	–	Alive (peritoneal metastasis 5 months postoperatively)
9	Ito S (2018) [22]	F	46	3.8	Y	N	–	Alive (10 months postoperatively)
10	Our case (2021)	F	75	11.5	Y	N	Negative	Alive (9 months postoperatively)

## Conclusions

 Extraskeletal osteosarcoma is a relatively uncommon soft tissue sarcoma, especially originating in the mesentery. ESOS growth in the abdominal cavity is relatively insidious, exhibiting typical clinical symptoms. Concurrently, the imaging features of ESOS are devoid of apparent characteristics. ESOS should also be considered when imaging reveals intraperitoneal solid-cystic or calcified masses. Its ultimate diagnosis depends on pathology. There is no agreement on the most effective treatment, and surgical excision is widely accepted. Chemotherapy remains widely controversial. Ensuring negative surgical margins may be an important factor affecting prognosis.

## Data Availability

Data sharing is not applicable to this article as no datasets were generated or analyzed during the current study.
